# Cost Assessment of Hepatitis B Virus-related Hepatitis in Bangladesh

**DOI:** 10.5005/jp-journals-10018-1190

**Published:** 2016-12-01

**Authors:** Mamun Al Mahtab, Muntasir Chaudhury, Mohammad H Uddin, Sheikh M Noor-E Alam, Mohammad A Rahim, Mohammad A Alam, Ahmed L Moben, Faiz A Khondaker, Mohammad FI Choudhury, Mohammad JA Sarkar, Provat K Poddar, Syed A Foez, Sheikh MF Akbar

**Affiliations:** 1 Department of Hepatology, Bangabandhu Sheikh Mujib Medical University,, Dhaka, Bangladesh; 2 Department of Economics, East West University, Dhaka, People’s Republic of Bangladesh; 3 Department of Medical Science, Toshiba General Hospital, Tokyo, Japan

**Keywords:** Bangladesh, Cost, Hepatitis B virus.

## Abstract

Hepatitis B virus (HBV) infection is endemic in Bangladesh. Studies have indicated that HBV is the major cause of chronic hepatitis B (CHB), liver cirrhosis (LC), and hepatocellular carcinoma (HCC) in this country. Recently, HBV-related acute on chronic liver failure (HBV-ACLF) has emerged as a serious and emergent medical problem in Bangladesh. To develop a strategy to address HBV-related problems and their influence on health care delivery system, proper understandings about extent of problems and nature of economic burden should be explored. Conservative estimates indicate that about 50 million or more of Bangladeshi have been infected by HBV at some point of their life. Out of the total Bangladeshi population, about 2 to 5% is chronically infected with HBV (about 3–8 million) (1–6%) and considerable number of these patients will eventually develop LC, HCC, or ACLF (about 1 million). Although proper statistics is lacking, it is estimated that HBV-related liver diseases account for a majority of hospital admissions and around 20,000 deaths every year in Bangladesh. In addition, complex clinical features of HBV-related liver diseases have been documented in Bangladesh that show similarity and differences from HBV infection in other Asian countries. Although vaccination against HBV and containment of horizontal transmission are in progress in Bangladesh for reduction of new HBV infection, there is a lack of national strategy for treatment of millions of chronic HBV-infected subjects. This paper will provide an insight regarding the economic impact of HBV in Bangladesh that may act as a primary impetus for developing national HBV eradication program, a goal set by World Health Organization (WHO).

**How to cite this article:**

Al Mahtab M, Chaudhury M, Uddin MH, Noor-E-Alam SM, Rahim MA, Alam MA, Moben AL, Khondaker FA, Choudhury MFI, Sarkar MJA, Poddar PK, Foez SA, Akbar SMF. Cost Assessment of Hepatitis B Virus-related Hepatitis in Bangladesh. Euroasian J Hepato-Gastroenterol 2016;6(2):163-166.

## EXTENT OF PROBLEM OF HBV IN BANGLADESH

As mentioned, HBV is highly prevalent in Bangladesh^1-6^ and about 3 to 8 million people of Bangladesh are chronically infected with the HBV as they allow HBV replication and express hepatitis B surface antigen (HBsAg). However, this is not only the major problem about HBV infection, rather this represents the tip of iceberg. About 25 to 30% of our population are also infected with HBV, but does not express HBsAg; rather express another marker of HBV, anti-HBc.^5,7^ Thus, about 50 million Bangladeshi are infected with HBV at some point of their lives and although they harbor low levels of HBV, transmission of their blood and body fluids may transmit HBV to healthy HBV-uninfected persons. Thus, HBV bears different faces of infection and studies have unveiled that the clinical presentation of HBV is extremely complex in Bangladesh and several million people are unaware of their HBV infection even though they have developed complications of liver diseases.^8-13^ These facts have multivariate influence on health care delivery system of this country in both short-term and also in the long-term.

In this communication, a gross account about direct economic impact related to HBV-related liver diseases will be provided and indirect and long-term impacts of HBV will not be at the center of this article, as we are now retrieving data to address indirect economic burden that arise from HBV.

## ECONOMIC IMPACT OF HBV; A CONSERVATIVE ASSESSMENT OF HBV-RELATED CHRONIC HEPATITIS B

**Fig. 1:**
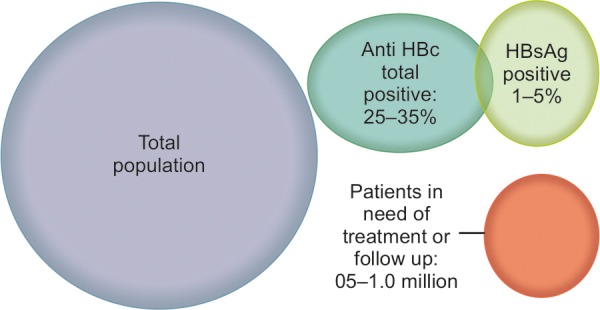
Magnitude of HBV-related liver diseases

Out of 3 to 8 million chronic HBV carriers, about 15 to 20% amounting to about 0.5 to 1 million should be treated because they have already developed CHB or are on the way of developing CHB, a condition in which HBV replication is associated with visible liver damages (Fig. 1). Considerable numbers of these patients with CHB would eventually develop LC and HCC or ACLF. The management of LC, HCC, and ACLF is highly costly and finally the outcome is extremely bad. Thus, treatment should be provided to CHB patients, so that they do not develop complications.

## TREATMENTS AND INVESTIGATIONS – TOTAL COSTS

With extensive worldwide research on HBV, multivaried scopes of investigations and options of treatments are currently available in both public and private hospitals. The cost of investigations to diagnose the presence of the virus mainly comes in two options: At baseline during first appearance or diagnosis and during follow-up period. The follow-up period may be highly variable based on the nature of disease state and future prognosis. The cost of assessment of HBV DNA, HBsAg, HBeAg, ALT and imaging of patients would cost about USD 200 during the initial diagnosis. If these investigations are accomplished in about one million prospective patients for 5 to 10 times during their lifetime, the economic cost would be about one billion USD. The cost to ensure HBV-free blood transfusion in Bangladesh would add to a very high price tag of additional cost that would not be discussed in this communication. It is presumed that abdominal imaging would be required in about 20% of these patients and that would cost about 20 million USD. Thus, direct cost related to bringing all prospective patients to initial diagnosis would be more than one billion USD in addition to substantial cost of patients screening, hospital attendance, and surveillance studies that may cost additional one billion USD.

**Fig. 2:**
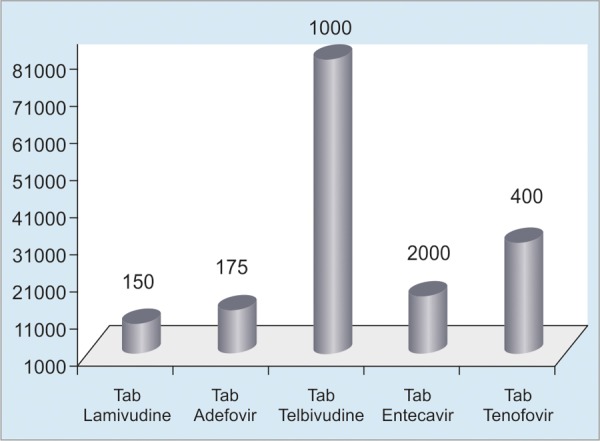
Costs of tablets over a period of 1 year

For curative measures, oral administrations include mainly five different kinds of medications that should be given for 5 to 10 years. Tablet entecavir and tablet tenofovir are most effective and extensively used. The cost of drugs for 1 year in USD has been shown in Figure 2.

Other than the abovementioned oral drugs, pegylated interferon (IFN) is also prescribed for a course of 48 weeks in injection form and costs about USD 6000 for one course. We now proceed to calculate the minimum total costs, which is the lowest priced tablet along with minimum time of treatment. Even if the 50% of prospective patients with CHB are brought under treatment, the lowest cost by cheapest oral drugs would be about 75 million/year and the total cost for an average therapy of 6 years would be about 450 million USD. The cost of usage of pegylated IFN would about USD 3 billion for 50% of prospective treatment group.

In addition to patients with CHB, more intensive treatment would be required for patients with LC and HCC. Sophisticated investigations would be required for their diagnosis and follow-up. Also, costly therapy would be required for their management. The cost of investigation and follow-up of therapy for LC patients varies from USD 3,000 to USD 10,000 and those of HCC patients may be amounting to USD 10,000 to USD 50,000.

The per-capita income of Bangladesh as per World Bank data is about USD 1100 (2011–2016). There are few public facilities at Bangladesh that can potentially provide therapy for CHB patients. Treatment of LC and HCC may be given in fewer public medical facilities. There is no national health care insurance system in Bangladesh that provides full support to therapy even in terminal stages of the diseases. Thus, the major bulk of therapies for patients of liver diseases are conducted in private facilities. Although some public health facilities have recently been developed, those are extremely limited considering the need of the country. In fact, the health care delivery system of Bangladesh is almost similar to most of the developing and resource-constrained countries of Asia and Africa. If the per-capita income of normal Bangladeshi patients are compared in the context of high cost of diagnosis, therapy, and follow-up of HBV-related liver diseases, it is apparent that comprehensive planning is needed as to how the cost of diagnosis can be reduced and how new drugs with cheaper price and finite therapeutic regimen may be materialized. Definitely, perception about HBV and its effect need to be known to the general population by dissemination of knowledge, change of attitude, and their reflection in performance.

## COMPILATION

The cost of management of HBV-related liver diseases would reach to several billion USD in Bangladesh and that is not a realistic option at this point in this country. In fact, studies from China and Iran have also predicted huge economic burden in the respective countries.^14-16^ We have only provided rough estimates of different facts related to HBV and its pathologies. In real-world situation, several more billion USD would be required to tackle the problem of case finding, training of manpower, and proper surveillance of patients. These facts indicate that there is need to develop a strategy of preference of therapy. Based on the economic condition of Bangladesh, a design of therapy should be developed and recommended, so that minimum investment can cause maximum output. The strategy is yet to be developed, but a comprehensive action of physicians, government, policymakers, politicians, patients, and representatives of civil society would provide their input and that may help to address these problems in an amicable manner. In addition to these direct costs, the amount of money needed for addressing social factors of HBV is also immense.^17,18^ However, we need to start an assessment of these factors, otherwise more complicated situations would be prevailing for decades and that cannot be addressed in centuries for the “terrorist virus” – HBV.
